# Seltene Differenzialdiagnose bei osteolytischer Läsion des Unterkiefers einer jungen Erwachsenen

**DOI:** 10.1007/s00292-024-01321-w

**Published:** 2024-04-11

**Authors:** Hyunkyu Shin, Andreas Naros, Sinja Kieninger, Joachim Polligkeit, Falko Fend, Jakob Milla

**Affiliations:** 1 Institut für Pathologie und Neuropathologie, Universitätsklinikum Tübingen, Tübingen, Deutschland; 2 Klinik und Poliklinik für Mund‑, Kiefer- und Gesichtschirurgie, Universitätsklinikum Tübingen, Tübingen, Deutschland

**Keywords:** ALK-positive Histiozytose, ALK-Histiozytose, ALK, Histiozytose, Knochenläsion, ALK-positive histiocytosis, ALK-related histiocytosis, ALK-rearranged histiocytosis, ALK, Histiocytosis

## Abstract

Wir stellen einen seltenen Fall mit hämatologischer Neoplasie bei einer jungen Erwachsenen vor, welche zuerst 2008 beschrieben wurde und seit 2022 in beide gängigen Tumorklassifikationssysteme hämatologischer Neoplasien, ICC und WHO, aufgenommen wurde. Diese Neoplasie zeigt eine charakteristische immunhisthochemische ALK-Positivität und entsprechend molekular ein ALK-Fusionsgen. Patholg*innen sollten diese Entität kennen, zumal eine Unterscheidung zwischen dieser Erkrankung und anderen häufiger auftretenden Erkrankungen des gleichen Formenkreises sowie einer mesenchymalen Neoplasie mit ALK-Aberration herausfordernd ist.

## Abstract (en)

We report a rarely occurring hematologic neoplasm in a young adult. Hematologic neoplasms were first described in 2008 and are now included in both accepted tumor classification systems, i.e., International Consensus Classification and World Health Organization. This hematologic neoplasm shows a characteristic ALK positivity in immunohistochemical examination and correspondingly, ALK fusion genes in the molecular analysis. Pathologists should be aware of this entity, particularly as it is challenging to differentiate from other more frequent neoplasms of the same disease group or mesenchymal neoplasm with ALK aberration.

## Anamnese

Eine 22-jährige Patientin stellt sich mit einer seit einigen Wochen bemerkbaren Einsenkung der Mundschleimhaut auf der linken Innenseite des Unterkiefers vor, die lediglich beim Kauen Beschwerden mache. Es bestehen keine hämatologischen Symptome wie Anämie, Infektanfälligkeit oder Blutungsneigung.

## Klinischer Befund

**Abb. 1:**
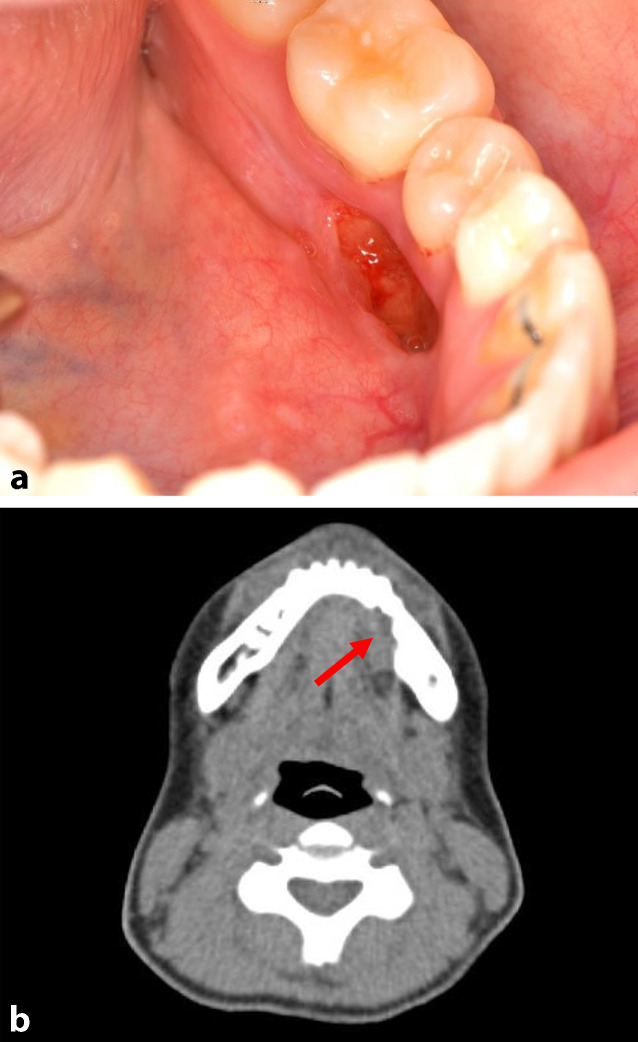
**a** Im Mundraum erkennt man eine 2 cm durchmessende, scharf begrenzte, ulkusartig eingesunkene Läsion auf der linken lingualen Seite des Alveolarkamms in Regio 35–37. **b** Im CT-Bild zeigt sich im korrespondierenden Bereich ein osteolytischer Befund mit stark unregelmäßiger Knochenkontur (*Pfeil*)

Man erkennt eine scharf begrenzte, tief reichende, ulzeröse Läsion auf der lingualen Lamelle des linken Unterkiefers in Regio 35–37 (Abb. 1) mit flächigem Granulationsgewebe, jedoch keinem freiliegenden Knochen. In der klinischen Untersuchung zeigt sich die Sensibilität und Motorik unauffällig. Die Sensibilitätsprüfung der Zähne ist ebenfalls unauffällig. Bei der Durchführung einer Probenentnahme zeigt sich der Knochen in diesem Bereich osteolytisch mit gelockerten Zähnen 35 und 36 sowie ausgefüllt mit gelblichem Material.

## Histologischer Befund

**Abb. 2:**
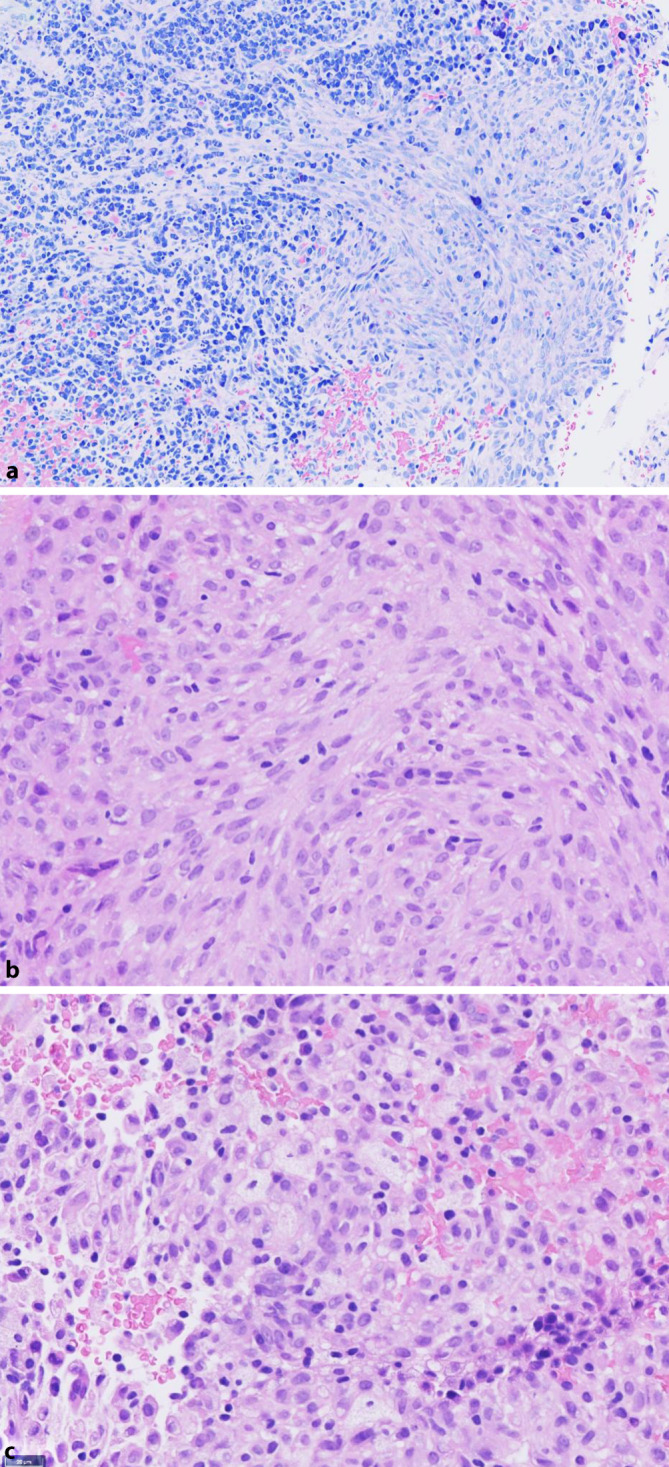
**a** Bezug vom läsionalen histiozytären Infiltrat zur ausgeprägten, periläsionalen, lymphoplasmazellulären Entzündung (Giemsa, 20 ×) . **b** In der starken Vergrößerung zeigt sich der eher spindelzellige Charakter der Histiozyten mit schmalem eosinophilem Zytoplasam mit „strömendem“ Muster (H.E., 40 ×). Vereinzelt sieht man in **c** auch reife (epitheloide) Histiozyten mit großleibigem, schaumigem Zytoplasma (H.E., 40 ×)

Man erkennt fragmentiertes Material aus dem Mundraum, teils überkleidet von reaktiv verändertem Plattenepithel mit Akanthose und elongierten, teils konfluierenden Reteleisten mit dem Aspekt einer pseudoepitheliomatösen Hyperplasie. Im Hintergrund zeigt sich ein lymphoplasmazelluläres Infiltrat mit Ausbildung von Granulationsgewebe sowie neutrophiler Entzündungskomponente, jedoch keine signifikante Eosinophilie. Auffallend ist ein submukosales, monomorphes, überwiegend spindelzelliges, teils epitheloides Proliferat in solider Anordnung teils mit angedeutet wirbeligem Muster. Die Zellkerne sind 2‑ bis 3‑mal größer als umliegende Lymphozyten, oval bis länglich, teils reniform mit Einkerbung. Das Chromatin ist zart und gleichmäßig verteilt, teils mit winzigen Nukleoli. Kaum mitotische Aktivität (1 Mitose/10 HPF; Gesichtsfelddurchmesser 0,5 mm). Das Zytoplasma ist mäßig breit und eosinophil. Außerdem eingestreut sind Schaumzellen mit reichlichem granulärem Zytoplasma. Das Zellproliferat infiltriert teils bis direkt unterhalb des Oberflächenepithels, ohne Infiltration des überkleidenden Epithels (Abb. 2).

**Abb. 3:**
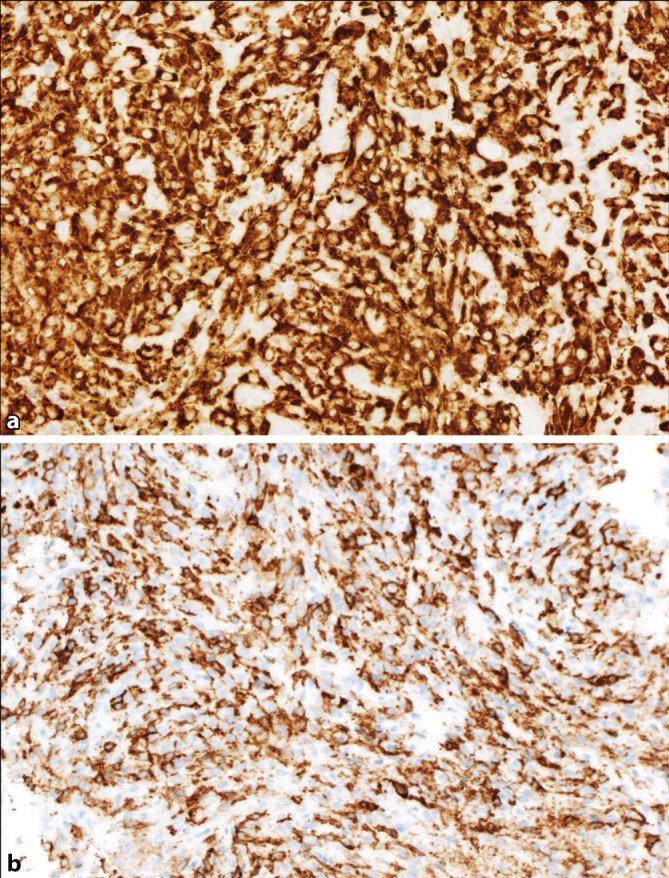
**a** Per definitionem zeigt die ALK-positive Histiozytose eine kräftige, zytoplasmatisch-membranäre Positivität für ALK (Roche D5F3). **b** In der Färbung für CD14 als monzytärem Marker zeigt sich eine deutliche Positivität der neoplastischen Histiozyten

Immunhistochemisch sind die neoplastischen Zellen positiv für CD68, heterogen für CD163, CD4, CD14 sowie CD45 mit unterschiedlicher Färbeintensität. Ein weiterer monozytärer Marker, Lysozym, bleibt negativ, ebenso wie S100 und CD1a. Die Färbung für ALK (Roche D5F3) zeigt eine kräftige, granulär-zytoplasmatische Positivität (Abb. 3). Weitere immunhistochemische Marker sowie molekulare Untersuchungen zum Ausschluss der in Frage kommenden Differenzialdiagnosen wie ein solitärer fibröser Tumor (STAT6), ein inflammatorischer myofibroblastärer Tumor (sm-Aktin) und eine noduläre Fasziitis (*USP6*-Translokation in der FISH) bleiben negativ.

Zur Detektion möglicher Genfusionen wurde eine NGS-Sequenzierung durchgeführt. Hierzu wurde RNA aus Formalin-fixierten Paraffinschnitten mit dem Maxwell® RSC RNA FFPE Kit und dem Maxwell® RSC Instrument (Promega, Madison, WI, USA) gemäß den Anweisungen des Herstellers extrahiert. Zur Analyse von Fusionsgenen wurde eine gezielte Multigenmutationsanalyse von 63 Genen mit dem Archer^TM^ FusionPlex^TM^ Sarcoma v2 Kit (ArcherDX, Integrated DNA Technologies, Coralville, Iowa, USA) mittels Next Generation Sequencing (Ion GeneStudio S5, Thermo Fisher Scientific, Waltham, MA, USA) nach Angaben der Hersteller durchgeführt. Die Archer^TM^ FusionPlex Technologie basiert auf der sog. Anchored Multiplex Polymerasekettenreaktion (AMP^TM^), welche den Nachweis bekannter und neuer Fusionspartner ermöglicht. Die Analyse und Interpretation der Sequenzierdaten erfolgte mit der Archer^TM^ Analysis v6.2.7 Software und ergab den Nachweis des Fusionsgens *EML4*::*ALK *(chr2:42472827, chr2:29446394); *EML4* (Exon 2, NM_019063.4), *ALK* (Exon 20, NM_004304.4) mit den für die Archer-Analyse relevanten Parametern: Unique Start Sites: 106, Reads: 447 und %Reads: 100.

## Diagnose

- ALK-positive Histiozytose, uniläsional.

## Therapie und Verlauf

Nach histologischer Sicherung des Befunds erfolgten die weitere hämatologische Abklärung und Durchführung eines PET-CT zum Ausschluss weiterer Manifestationen.

Bei einem umschriebenen Tumor im Sinne einer Singlesystemhistiozytose wurde ein operatives Vorgehen mit R0-Resektion angestrebt. Hierfür erfolgte in ambulanter Narkose die Extraktion der Zähne 34 und 35 mit Kürettage und anschließendem Ausfräsen und modellierender Osteotomie der Osteolyse. Der weitere Verlauf gestaltete sich unauffällig, bei guter Wundheilung und ohne klinischen Verdacht auf ein lokales Rezidiv. Nach weiteren klinischen und radiologischen Kontrollen ist im tumorfreien Intervall eine Versorgung des Kieferabschnitts mit Zahnimplantaten angedacht.

## Diskussion

Unter einer Histiozytose im weiteren Sinne versteht man ein breites Spektrum von teils klonalen, teils reaktiven Proliferationen unterschiedlicher Histiozytentypen, d. h. gewebeständigen Makrophagen, dermalen/interstitiellen dendritischen Zellen oder Langerhans-Zellen unterschiedlicher Dignität, somit sehr variablem klinischem Verhalten. Die Klassifikation der Histiocyte Society unterteilt Histiozytosen je nach klinischen, histologischen und molekularen Charakteristika in fünf Gruppen, nämlich L‑ (*L*angerhans-related), C‑ (*c*utaneous and mucocutaneous), R‑ (*R*osai-Dorfman disease), M‑ (*m*alignant) und H‑Gruppe (*h*aemophagocytic) [1].

Die ALK-positive Histiozytose ist eine sehr seltene histiozytäre Neoplasie, die in 2008 zum ersten Mal in drei Säuglingen beschrieben wurde und mittlerweile in den beiden gängigen Klassifikationssystemen hämatologischer Neoplasien (International Consensus Classification von 2022 und WHO-Klassifikation HAEM5 von 2022) im Kapitel histiozytärer Neoplasien aufgenommen worden ist [2, 3, 8]. Durch darauffolgende weitere Berichte und Metaanalysen wurden klinisch-pathologische Merkmale dieser neuen Entität der Histiozytose näher charakterisiert, welche in nahezu jeder Lokalisation und jeglicher Altersgruppe auftreten kann [5, 6]. Entsprechend der Befallsmuster kann die ALK-positive Histiozytose gemäß der Arbeit von Kemps weiter in drei klinisch-phänotypische Gruppen eingeteilt werden: Gruppe IA Multisystemerkrankung mit Beteiligung von Leber und Hämatopoese, Gruppe IB Multisystemerkrankung mit Beteiligung anderer Organe, Gruppe II Singlesystemerkrankung [4].

H.E.-morphologisch können sich die läsionalen Zellen der ALK-positiven Histiozytose sowohl epitheloid mit reichlichem schaumigem Zytoplasma, als auch spindelzellig darstellen, und sie sind bevorzugt in dichten Zellrasen angeordnet, ohne Epidermotropismus. Die Kerne sind häufig reniform und weisen eine unregelmäßige Kernkontur mit Einkerbungen auf, allerdings ohne prominente Kernpleomorphie oder erhöhte mitotische Aktivität. Mehrkernige läsionale Zellen, wie Touton-Zellen, können beobachtet werden.

Immunhistochemisch sind die läsionalen Zellen aus der monozytär-histiozytären Linie variabel positiv für die entsprechenden Marker wie CD68, CD163, CD14, CD4 sowie Lysozym. S100 kann eine Positivität zeigen. Fascin und Faktor XIIIa sind auch häufig positiv. CD1a und Langerin, welche auf eine Langerhans-Zell-Histiozytose hinweisen, bleiben negativ. Per definitionem sind die läsionalen Zellen kräftig und überwiegend zytoplasmatisch positiv für ALK, wobei zu betonen ist, dass verschiedene Klone unterschiedlicher Hersteller, z. B. D5F3, 5A4, 1A4 und ALK1 zur Verfügung stehen und gemäß Expertenmeinung davon stets mehr als ein Klon zur Diagnose verwendet werden soll, um falsch-negative Ergebnisse zu vermeiden.

**Abb. 4:**
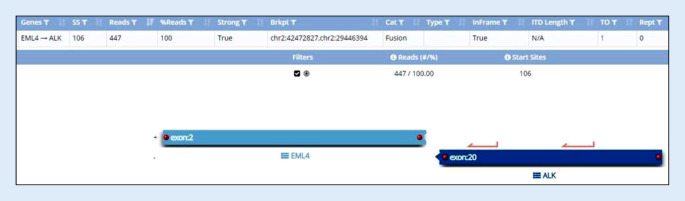
In der Next-generation-sequencing (NGS)-Analyse mittels Archer® FusionPlex® Sarcoma V2 Kit (Thermo Fisher Scientific, Massachusetts USA) wurde das Fusionsgen *EML4* (Exon 2)::*ALK* (Exon 20), beide auf dem Chromosom 2, detektiert

In der NGS-Analyse mittels Archer-Fusionspanel für den vorliegenden Fall wurde das Fusionsgen *EML4::ALK* nachgewiesen, was unsere Diagnose bestätigte (Abb. 4). Als Fusionspartner von *ALK* wurden zahlreiche weitere Gene identifiziert, unter denen *KIF5B* der häufigste Fusionspartner von *ALK* darstellt (je nach Studie, bis zu 83 % der Fälle; [7]). Die vom Adenokarzinom der Lunge bekannte *EML4::ALK*-Translokation wurde aber ebenfalls mehrfach in der ALK-positiven Histiozytose beschrieben. Das Fusionsgen führt zu einer dauerhaften ligandenunabhängigen Aktivierung der Rezeptortyrosinkinase (ALK) und deren Downstream-Signalwege, wie dem PIK3K-AKT-mTOR- oder dem RAS-RAF-MEK-ERK-Signalweg. Dadurch kommt es zu einer pathologischen Vermehrung des den Zellzyklus regulierenden Proteins Cyclin D1, welches die neoplastische Proliferation der läsionalen Zellen fördert.

**Abb. 5:**
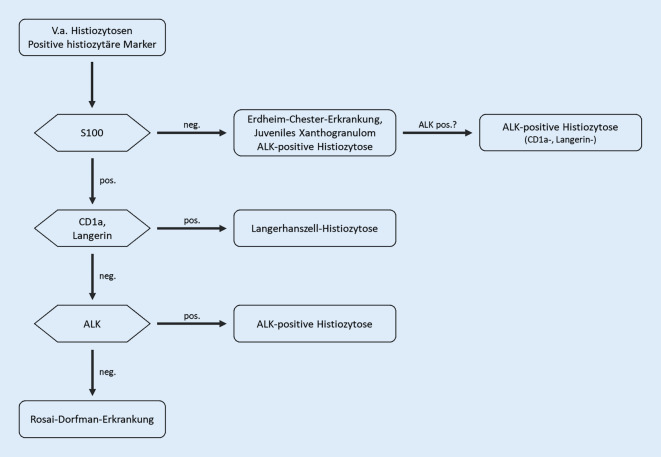
Vorschlag zu einem Flussdiagramm beim immunhistochemischen Befundvorgang der differenzialdiagnostisch infrage kommenden Histiozytosen. Die am Ende des jeweiligen Wegs stehenden Diagnosen müssen H.E.-morphologisch oder molekularpathologisch weiter differenziert oder bestätigt werden

Da die ALK-positive Histiozytose eine sehr seltene Erkrankung darstellt, müssen andere häufiger vorkommende Histiozytosen histomorphologisch und immunhistochemisch ausgeschlossen werden, z. B. Langerhans-Zell-Histiozytose und juveniles Xanthogranulom (Tab. 1 und Abb. 5). Unter Umständen kann auch eine mesenchymale Neoplasie, v. a. ein inflammatorischer myofibroblastärer Tumor (ebenfalls ALK+, jedoch sm-Aktin+), differentialdiagnostisch in Betracht kommen, zumal die Histomorphologie der ALK-positiven Histiozytose oft ein solides, wirbeliges Wuchsmuster von Spindelzellen zeigt. Wie andere ALK-rearrangierte Erkrankungen, z. B. der inflammatorische myofibroblastäre Tumor, wird die ALK-positive Histiozytose vorrangig bei weiblichen Individuen beobachtet [4].

**Tab. 1:** Hier sind naheliegende Differenzialdiagnosen zur ALK-positiven Histiozytose mit deren immunhistochemischem und molekularem Profil sowie diagnostisch wegweisender wichtiger H.E.-Morphologie aufgeführt. Diese Histiozytosen zeigen ebenfalls grundsätzlich eine Positivität für histiozytäre Marker wie CD68, CD163, CD4, CD14, Lysozym

Differenzialdiagnosen	Immunhistochemisches Profil	Weitere diagnostische Kriterien
Langerhans-Zell-Histiozytose	S100+, CD1a+, Langerin+	Eosinophilie im Hintergrund, häufig *BRAF*-Mutation
Rosai-Dorfman-Erkrankung	S100+, CD1a−, Langerin−, OCT2+	Prominente Emperipolese, gelegentlich *KRAS-* und *MAP2K1-*Mutationen
Erdheim-Chester-Erkrankung	S100−, CD1a−, Langerin−, OCT2−	Eher hohes Erkrankungsalter, häufig *BRAF*-Mutation
Juveniles Xanthogranulom	S100−, CD1a−, Langerin−	Eher niedriges Erkrankungsalter (Kinder), keine *BRAF*-Mutation
ALK-positive Histiozytose	S100+/−, CD1a−, Langerin−, Faktor XIIIa+, Cyclin D1	*ALK*-Translokation (am häufigsten mit *ALK::KIF5B*)
Inflammatorischer myofibroblastärer Tumor	ALK+, SMA+, negative histiozytäre marker	Ggf. vermehrte Histiozyten im Hintergrund

Die Prognose der ALK-positiven Histiozytose ist trotz deren offensichtlich neoplastischer Genese (ALK) generell gut, und so wird in der Literatur eine spontane Remission unter alleiniger supportiver Therapie berichtet; in anderen Fällen ist eine Behandlung mit ALK-Inhibitoren indiziert, was eine definitive korrekte Diagnose dieser Erkrankung, v. a. zur Abgrenzung von häufigeren Histiozytosen erforderlich macht. Die bei der Langerhans-Zell-Histiozytose gut bekannten, prognoseverschlechternden Faktoren wie Multisystemerkrankung oder Beteiligung der Risikoorgane (Leber, Milz, Hämatopoese) scheinen bei der ALK-positiven Histiozytose nicht unbedingt mit schlechtem Verlauf assoziiert zu sein. Es gab allerdings auch Einzelfälle mit letalem Krankheitsverlauf [4].

## Fazit für die Praxis

- Zusammenfassend sollte insbesondere bei CD1a- und Langerin-negativen Histiozytosen eine ALK-Färbung durchgeführt werden.
- Ein positives Ergebnis sollte durch eine weiterführende NGS-Analyse bestätigt werden, um Patient:innen bei Bedarf nicht eine klinisch hoch wirksame ALK-Inhibitionstherapie vorzuenthalten.
